# Strategies for the Analysis of Large Social Media Corpora: Sampling and Keyword Extraction Methods

**DOI:** 10.1007/s41701-023-00143-0

**Published:** 2023-04-30

**Authors:** Antonio Moreno-Ortiz, María García-Gámez

**Affiliations:** Department of English, French and German Philology, University of Málaga, Málaga, Spain

**Keywords:** Covid-19 language, Large-scale social media corpus, Sampling methods, Sampling sizes, Keyword extraction

## Abstract

In the context of the COVID-19 pandemic, social media platforms such as Twitter have been of great importance for users to exchange news, ideas, and perceptions. Researchers from fields such as discourse analysis and the social sciences have resorted to this content to explore public opinion and stance on this topic, and they have tried to gather information through the compilation of large-scale corpora. However, the size of such corpora is both an advantage and a drawback, as simple text retrieval techniques and tools may prove to be impractical or altogether incapable of handling such masses of data. This study provides methodological and practical cues on how to manage the contents of a large-scale social media corpus such as Chen et al. (JMIR Public Health Surveill 6(2):e19273, 2020) COVID-19 corpus. We compare and evaluate, in terms of efficiency and efficacy, available methods to handle such a large corpus. First, we compare different sample sizes to assess whether it is possible to achieve similar results despite the size difference and evaluate sampling methods following a specific data management approach to storing the original corpus. Second, we examine two keyword extraction methodologies commonly used to obtain a compact representation of the main subject and topics of a text: the traditional method used in corpus linguistics, which compares word frequencies using a reference corpus, and graph-based techniques as developed in Natural Language Processing tasks. The methods and strategies discussed in this study enable valuable quantitative and qualitative analyses of an otherwise intractable mass of social media data.

## Introduction

Social media has become one of the main resources for researchers in many fields where public opinion, attitudes, and perceptions are relevant, such as discourse analysis and the social sciences, which seek to study how communication takes place among people in a globalized world. As the use of online platforms has generalized as a means to express and exchange ideas, researchers have turned to this content to explore public opinion and stance on specific social topics, as well as the language they use. This is specifically relevant if we bear in mind the context of the past two years, characterized by one of the worst health crises in contemporary history.

The language of the COVID-19 pandemic has been studied from different perspectives and with different operational and cultural contexts in mind. From a linguistic perspective, scholars have made a tremendous effort to gather information in public access repositories, such as the Oxford Covid-19 language hub (Oxford Languages, 2022). Several large-scale corpora have been made available to the scientific community, such as the CORD-19 Corpus (Wang et al., 2020), which comprises scientific academic publications, or the Coronavirus Corpus (Davies, 2021), composed of news items from online newspapers and magazines from 20 different English-speaking countries.

The analysis of social conversation has also been essential, particularly at a time when people were forced to stay at home and turned to social media to express their feelings. Of such platforms, Twitter stands out as one of the most relevant in research, as it presents some key features that differentiate it from other forums, namely: (i) its conciseness; (ii) its anonymity, which allows speakers to express their ideas without fear of being identified; and (iii) the nature of the posts, which constitute what is known as user-generated content (UGC), with a number of important idiosyncrasies. In addition, this type of content includes high velocity granular data which includes metadata that allows the analysis of a phenomenon’s evolution over time (Lee & Yee, 2020).

Twitter data have been used to carry out research around the COVID-19 pandemic with a myriad of methodologies and objectives: among others, Mackey et al. (2020) use Twitter data to research symptoms associated with the disease, Pulido et al. (2020) pay attention to the spread of false information, and Ferrara (2020) focuses on the role played by bots in such process.

Multiple available COVID-19 Twitter datasets have been compiled and made available to the academic community (Banda et al., 2020; Dimitrov et al., 2020; Lamsal, 2021), but the corpus created by Chen et al. (2020) stands out as the largest, both in terms of size (with over 31 billion words) and time span, as the data were collected from January 21, 2020, and the process is still ongoing. However, the size of this corpus is both an advantage and a drawback, as it requires users to implement their own Natural Language Processing (NLP hereafter) techniques if they wish to analyze big data, as manual, qualitative analysis is simply unfeasible. The problem is that such techniques are often computationally intensive and difficult to learn, which usually becomes a limitation for researchers. Moreover, desktop corpus tools such as *WordSmith* (Scott, 1996) and *AntConc* (Anthony, 2022), or web-based tools that allow uploading user corpora, such as *SketchEngine* (Kilgarriff et al., 2014*)*, simply cannot handle such massive amounts of text, as they do not have text-indexing capabilities (in the case of desktop applications), or do not allow uploading such large amounts of text. Therefore, it is necessary to come up with suitable methodological underpinnings, as well as specific strategies that facilitate managing and exploring such large-scale UGC corpora.

Thus, in this work we aim to provide methodological and practical cues on how to manage and explore the contents of a large-scale social media corpus such as Chen et al. (2020)’s Covid-19 corpus. The main objective is to compare and evaluate, in terms of efficiency and efficacy, available methods to handle large-scale social media corpora. In this way, this study leverages and compares the use of different methods and approaches. First, we aim to compare the use of differing sample sizes to assess whether it is possible to achieve similar results despite the size difference, and to evaluate sampling methods such as proportional-to-size sampling (PPS) following a specific data management approach to storing the original corpus. Second, this work will examine two keyword extraction tools that have different methodological approaches to the process: the traditional method used in corpus linguistics, which employs a reference corpus to compare word frequencies using a range of different statistical measures, and graph-based techniques as developed in NLP applications. These objectives are tackled using an experimental methodology, and evaluation of results will be performed employing specific formal metrics where possible, as assessing keyword extraction performance or quality is prey to subjective interpretation (Gabrielatos, 2018).

## Sampling Methods and Keyword Analysis

This section aims to provide an overview of the literature regarding the main topics in our research: sampling size and methods, as well as keyword extraction and analysis.

### Sampling

As stated by Boyd and Crawford (2012), “just because Big Data presents us with large quantities of data does not mean that methodological issues are no longer relevant. Understanding sample, for example, is more important now than ever” (p. 668). When analyzing data extracted from Twitter, and working with such a large corpus as the one compiled by Chen et al. (2020), one of the most important aspects to keep in mind is sampling, since trying to analyze the whole corpus is either impractical or not possible altogether. Twitter data basically consists of a large amount of small, similar texts, many of which are simply a repetition of each other (retweets). For this reason, preparing the data and using a consistent sampling method, as well as a representative sample size, is essential, as it can greatly optimize data storage and processing.

Sampling is the set of methods whereby a subset of units is selected from the target population. Defining the population to sample is not an easy task, yet it is of paramount importance to avoid bias and to subsequently make accurate generalizations from the sampled data. This process can be split into two different designs, each with its own subset of methods: probability and non-probability sampling. The main feature that distinguishes these schemes is that the latter selects the units through a non-random, and thus subjective, method, while the principle of randomization is what characterizes the former. In this work we will focus on probability sampling, which includes, in turn, different sub-methods (Beliga et al., 2015; Siddiqi & Sharan, 2015): (i) simple random sampling, (ii) systematic sampling, (iii) stratified sampling, (iv) cluster sampling, (v) multistage sampling, (vi) multiphase sampling, and (vii) proportional-to-size sampling.

Of the aforementioned methods, two are relevant to our work: simple random sampling, which is the most generally used due to its simplicity; and proportional-to-size sampling, the one we employ in our study. Simple random only requires a list of all the units of the target population and all members of the population have the same probability of being drawn for the sample. However, as reported by Kamakura (2010), one of its drawbacks is that the random drawing may lead to the over- or underrepresentation of small segments of the population: since all of the members of the sampling frame can be randomly drawn, it leaves to fate to which extent a particular group will be represented—or if it is at all—in the sample. Kamakura explains this with an accurate example: imagine that we must carry out a study in which ethnicity is one of the relevant aspects to bear in mind and Asians represent 2% of the population. A sample of members randomly drawn from the population may include 5 Asians or no Asians at all, thus resulting in a representation issue. Therefore, ensuring representation is a task that may require more fine-grained sampling techniques beyond the principle of randomization, such as proportional-to-size sampling. This method requires a finite population of units, in which a size measure “is available for each population unit before sampling and where the probability of selecting a unit is proportional to its size” (Skinner, 2016, p. 1). Therefore, the chances of being included in the sample are bigger as the size of the unit increases. It is for this reason that the measure of size must be accurate.

### Sampling Versus Representativeness

In the context of linguistic studies based on the analysis of large collections of electronic texts, the issue of linguistic representativeness needs to be addressed in relation to sampling, as extracting a sample may impact the level of representativeness of the original corpus. Corpora are generally understood and used as a sample of a larger population, that is, the corpus itself is the sample, which attempts to represent a language as a whole or a specific domain, time period, register, etc. Thus, it is the corpus that must be representative, and this depends on the extent to which “it includes the full range of variability in a population” (Biber, 1993, p. 244). As explained by Clear (1992), in the process of designing a corpus it is important to consider the relationship between the sample and the target population, as “the distributional characteristics of items included in the sample should match those of the target population” (p. 24). However, the majority of the previous studies on representativeness in corpus linguistics have largely paid attention to the issue of sampling sizes, going as far as to state that size is the most relevant aspect to bear in mind in corpus design (Hanks, 2012).

Of course, the concept of representativeness itself is far from being uncontroversial. McEnery et al. (2006) point out two main concepts to be taken into account: (i) target domain, which determines whether the corpus is representative of the full range of text type variability existing in the target domain, and (ii) linguistic representativeness, which examines if the corpus contains the full range of linguistic distributions in the target domain. On a very recent elaboration on the concept, Egbert et al. (2022) summarize ten conceptualizations of what a representative corpus is, or should be, according to the scientific literature, which range from a general acclaim for data to more elaborate considerations regarding coverage or the absence of selective focus. The authors consider that domain considerations should rely on the qualitative characteristics of the domain to select what the corpus should contain, whereas distribution considerations should be quantitative and relative to the variation of linguistic features of interest.

The issue of representativeness has also been traditionally determined by sample size. A decade ago, Hanks (2012) still defended the relevance of larger corpora, as a consequence of the relationship between types and tokens, and suggested that the larger the corpus, the higher the possibility to distinguish “statistically significant co-occurrences of words from chance” (p. 403). However, this traditional “data-hungry” approach, embodied in the expression “there’s no data like more data^1^”, is now trumped by the generalized availability of very large datasets. In this day and time many corpora run into the billions of words.

In our case, the corpus compiled by Chen et al. (2020) contains over 31 billion words for English alone (as of December 2021) and was compiled using Twitter’s streaming API, which, by design, returns only 1% of the full set of tweets, as acknowledged by the corpus designers themselves (Chen et al., 2020, p. 6). Thus, the original corpus itself cannot be said to guarantee linguistic representativeness of the language used in online social media to talk about COVID-19, as this 1% sample of the total volume of tweets must be assumed to be random, not following any organization or distributional criteria. Then, if we are to follow the criterion that in order for a corpus to be representative it needs to include the full range of linguistic variability (see Biber’s definition quoted above), linguistic representativeness cannot be taken for granted. However, even this small percentage renders massive amounts of data, which would satisfy the, admittedly controversial, “data-hungry” view on representativeness. It is important to understand (and accept) that this lack of linguistic representativeness is true not only of this particular corpus, but of any social media corpus. Firstly, social media is controlled by the companies that provide the infrastructure and ultimately own the content and decide whether to make it available and under which terms. But more importantly, we need not forget the very nature of online user-generated content, where aspects like completeness, organization, or traceability are lost in favor of sheer volume.

More relevant to our objectives, however, is statistical representativeness. From this perspective, the question is: given a very large corpus (and regardless of whether it is linguistically representative), what sampling strategies can be used in order to make it manageable? In section "Corpus Sampling" we describe the strategies we propose.

## Keyword Analysis

Keywords are lexical items that accurately describe the main subject of a text and which are a compact representation of the document under study (Beliga et al., 2015). Keyword analysis methods can be divided into two broad categories: keyword assignment and keyword extraction. In the former, potential keywords are selected from a given, controlled set of words, while the latter pursues the identification of the most relevant words in a document (Onan et al., 2016). Both approaches, however, focus on the same problem: the selection of the most representative words and phrases.

Previous definitions of this term, nevertheless, are expressed in terms of the metrics proposed for their identification and extraction; for example, Scott (1996) stated that keywords are words “whose frequency is unusually high in comparison with some norm” (p. 53). This is a methodological rather than notional definition, and thus relies on the assumption that the only available method for keyword identification is using a reference corpus or a word frequency list, which is the traditional way in which keywords have been extracted in corpus linguistics. This method comes down to comparing word frequencies between the target (or *focus*) corpus and the corpus of reference, which is assumed to have a normal distribution of word frequency. Nevertheless, there are two issues with this definition: (i) it ignores linguistic features such as homography, polysemy, and syntactic relations, and (ii) it leaves out the existence of other multiple ways to identify keywords.

In addition to extracting what is special about a certain type of language, keyword identification can serve other more general purposes and reflect what a corpus is about, being thus an alternative or a complement to topic modeling. In fact, the extraction of keywords has become one of the most important tasks not only in text mining, but also in NLP and information retrieval in general (Beliga et al., 2015). Within the NLP arena, several approaches to keyword extraction have been proposed: (i) simple statistical, (ii) linguistic, (iii) machine learning (ML), and (iv) other (Zhang et al., 2008). Simple statistical approaches do not require training data and rely on simple methods that are language and domain independent. As reported by Beliga et al. (2015), the statistics of the words from a text can be used to identify keywords, such as n-gram statistics, word frequency and co-occurrences, or suffix trees. This method, nevertheless, also presents some disadvantages: for instance, in texts belonging to specific domains, relevant keywords often appear only once in the text and might thus be not identified as keywords.

Linguistic approaches are generally based on syntactic, lexical, semantic, and discourse features that are examined to extract the main keywords (Siddiqi & Sharan, 2015). On the other hand, machine learning approaches apply supervised or unsupervised learning techniques, although the former is preferred for keyword extraction. A drawback of this method is that it requires annotated training data sources, and it is domain-dependent, which entails that a new predictive model needs to be trained for each specific domain (from a new training dataset). Vector Space Models (VSM) have been also used for keyword extraction, as they are currently the most widely employed method for text representation. A VSM represents documents as feature vectors in a multidimensional Euclidean space, and although they are being successfully applied to a variety language processing tasks, they also have been shown to have disadvantages: (i) the semantics of a text are not explicitly expressed, (ii) words are independent from each other, and (iii) if two documents “have a similar meaning but they are of different words, similarity cannot be computed easily” (Sonawane & Kulkarni, 2014, p. 1).

Finally, graph-based text representations successfully address many of these issues (Sonawane & Kulkarni, 2014). In this approach, the document is represented as a graph, and since graphs are mathematical models, they enable the exploration of the relationships between words, so that they are not analyzed as independent from each other. The widely used *TextRank* algorithm (Mihalcea & Tarau, 2004) employs a graph-based ranking model and is language-independent. In this work, we will use *TextRank* to compare the results of graph-based keyword extraction with those of the traditional, statistics-based approach generally used in corpus linguistics, as exemplified by the online-corpus management and retrieval web application *SketchEngine* (Kilgarriff et al., 2014). Keyword extraction by this method relies on computing a *keyness* score for each word and limited-size n-gram in the corpus. Unlike other corpus management and retrieval applications, which offer several user-selectable statistics for keyword extraction,^2^*SketchEngine* computes this keyness score using the statistic known as *simple math* (Kilgariff, 2009). Other statistics have been claimed to offer better performance (Gabrielatos, 2018), but *SketchEngine* is possibly the most widely used corpus management and retrieval platform and therefore will serve us as the perfect illustration for our purposes.

## Method

### Corpus

We use the corpus developed by Chen et al. (2020), an ongoing collection of tweets related to the COVID-19 pandemic^3^. The authors used Twitter’s API and the Tweepy Python library to compile tweets since January 21, 2020. The searches were done using specific accounts and keywords that, at the time, were trending (e.g., “coronavirus”, “corona”, “COVID-19”). While the dataset contains tweets in over 67 languages, Chen et al. (2020) do admit that there is a significant bias towards English tweets over other languages.

This corpus has already been used in previous research: among others, Bahja and Safdar (2020) carried out sentiment analysis and topic modeling through clusters, Aiello et al. (2021) tested Strong’s model and analyzed the corpus thematically using NLP and ML techniques, and Li et al. (2021) assessed how non-governmental organizations use Twitter to form communities and to address social issues. To our knowledge, no previous studies have centered on the methodological underpinnings of using such a large-scale social media corpus as this one is.

**Fig. 1:**
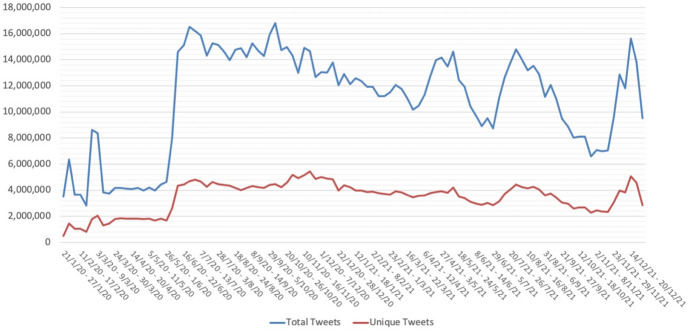
Tweets processed and unique over time (aggregated by week)

Chen et al.’s corpus is not without its shortcomings: the authors acknowledge that there are some known gaps in the dataset because of Twitter API restrictions on access to data and the collection of data through the leveraging of Twitter’s streaming API, which only returns 1% of the total Twitter volume, so the number of collected tweets depends on their network connection and their filter endpoint. Also, the list of keywords used for the streaming API was modified and expanded as related terms (e.g., “lockdown”, “quarantine”) emerged, which explains the sudden leaps in the number of tweets at certain points (see Fig. 1).

The original corpus is distributed as a set of *gzipped* text files that contain the IDs of every tweet contained in the corpus, along with a Python script (“hydrate.py”) that downloads the tweets using Twitter’s streaming API. It takes an average of 12 days-download time for every month in the corpus, as Twitter imposes certain bandwidth limits, and the download process needs to be paused at regular intervals. The corpus is then downloaded as a series of *gzipped* JSON lines files, where each JSON line is a tweet. These files contain all tweets for all languages, and each tweet contains the full tweet data.

Therefore, the first step is to extract the tweets themselves from the original files. We used a custom Python script to process the downloaded tweets and extract the English ones, keeping only certain data for each tweet (tweet ID, user, date, and text). A key feature of our tweet extraction method is that we avoid saving retweets and repeated tweets and save only one instance of every tweet per day, along with a counter indicating the number of times that such tweet occurs in the day set. Thus, we added a *n* datum to each tweet indicating its (daily) frequency. During this tweet extraction process we also pre-processed the text to remove hyperlinks and certain characters such as newlines, tabs, and Unicode characters known to cause issues (e.g., typographic quotes). We also filtered out tweets shorter than 3 words. This procedure allows us to optimize storage and, more importantly, processing time. Figure 1 shows the number of tweets processed and kept over time at week intervals. We provide specific counts in section "Corpus Sampling".

In the original corpus there is one file for each hour of each day. During the extraction process by language, we merged all unique tweets for one day into one file. Therefore, we ended up with as many files as days in the corpus, and each line in the files is one tweet. All files were compressed using *gzip*, as this compressed format can be uncompressed on-the-fly while opening them. An example of this output is given in (1):

1. {“text”: “BEWARE! THE CHINA VIRUS HAS LANDED IN US! CDC Confirms First U.S. Case of Deadly China Virus. via @BreitbartNews”, “user”: “trumptrain1111”, “date”: “Tue Jan 21 19:58:49 +0000 2020”, “id”: “1219710963617861632”, “n”: 4}

The corpus was not lemmatized for any of the tasks described. The reason for this decision is that the TextRank algorithm works by creating graphs of syntactic patterns, which heavily relies on accurate part-of-speech tagging, which in turn requires that the original word forms be present.

### Objectives

The general objective of this work is to provide methodological cues and strategies on how to manage and explore the contents of large-scale social media corpora. This general objective encompasses two operational requisites that determine the specific objectives, as detailed below:

- Specific objective 1: To compare different sampling sizes to decide which one optimally represents the whole corpus for keyword extraction while keeping data to a manageable size using accessible^4^ computing resources.
- Specific objective 2: To compare two different methods of keyword extraction: the reference-corpus method commonly used in corpus linguistics and the graph-based method commonly used in NLP.

### Corpus Sampling

Here we explore and test the hypothesis that working with smaller, fixed-interval samples is practical and efficient, and that the results obtained for certain tasks, such as keyword extraction, are similar to using bigger samples or even the full corpus. Table 1 summarizes the number of tweets and tokens contained in the full corpus and in each of the samples we extracted.

**Table 1:** Basic statistics of English corpus and samples

	0.1% Sample	0.5% Sample	1% Sample	Full corpus
N tweets (actual)	923,550	3,940,969	7,245,394	352,556,633
N tweets (rep.)	1,104,964	5,526,188	11,052,737	1,117,379,746
N tokens (actual)	28,754,912	109,303,100	199,369,396	9,134,879,457
N tokens (rep.)	31,236,676	156,348,389	312,746,780	31,292,640,403
Space saving^a^	16.42%	28.69%	34.45%	68.45%

^a^Space saving is calculated with the equation $1-\frac{\mathrm{Compressed\, Size}}{\mathrm{Uncompressed\, Size}}$, expressed as a percentage and using the number of tweets.

To extract these samples, we regard the corpus as a time series of day intervals. Our sample extraction script takes the desired percentage of the corpus to include in the sample and extracts a daily, randomized, proportional-to-size sample. Proportionality is based on the frequency information that we obtained during the tweet extraction process. Like the full corpus, samples are stored as *gzipped* JSONL files (one file per day, one JSONL document per tweet), where each JSONL document includes the tweet’s text, date, and its frequency. With this system, we save considerable processing time. Thus, instead of processing the actual number of tweets (many of which are the same text because they are retweeted or copy-pasted), we can simply use the tweet’s frequency as a factor to multiply results by. Table 2 summarizes the processing times for the most computing-intensive operations, including the sample extraction and the keyword extraction process.

**Table 2:** Processing times of the most computing-intensive operations

Task description	Sample	Time taken
0.1% Sample extraction	Full corpus	00:58
0.5% Sample extraction	Full corpus	01:03
1% Sample extraction	Full corpus	01:05
Keyword extraction	0.1%	05:57^a^
Keyword extraction	1%	48:32^a^

All tasks were run on an Intel Core i7 -7400 3.0 GHz CPU (4 cores) on Ubuntu Linux 20.04 Server 64-bit. We have only included those tasks that took a significant amount of time. All times are given in hh:mm format.

^a^During the keyword extraction process, we also filtered candidate keywords and extracted other relevant text items (entities, mentions, hashtags, and emojis), thus adding considerable processing time.

As shown in Table 2, although the sample extraction time is similar for the 0.1%, 0.5%, and 1%, sample size becomes an important factor in the keyword extraction task: in the case of the 1% sample, this task alone took over 48 hours, in comparison to the 5 hours needed for the 0.1% sample; this is the main reason that led us to research whether working with a smaller sample (0.1%) could be a good option to obtain similar results.

## Keyword Extraction

We employ two different methods of keyword extraction, described in section "Sampling Versus Representativeness" above, to compare results. On the one hand, we use a graph-based keyword extraction method and, on the other, we extract keywords with *SketchEngine* (SE), which uses a statistics-based approach. We run both methods on two different sample sizes (0.1% sample and 1% sample) to decide whether processing a substantially larger sample is worth the considerable processing overhead.

Although we aim to focus on keywords, our script for graph-based text representation, *TextRank*, also extracts entities, hashtags, and emojis. It uses the SpaCy (Honnibal et al., 2020) NLP toolkit with two specific pipeline extensions: PyTextRank (Nathan, 2016) to extract keywords, and Spacymoji^5^ to extract emojis. Entities are extracted using spaCy’s built-in entity recognition features and hashtags are identified using regular expressions. The script builds dictionaries of each of these categories, where each entry has frequency information in all cases. Keywords also have a rank score (akin to a keyness score), as returned by PyTextRank.

Running spaCy on millions of tweets one by one is an extremely slow task, because one spaCy *doc* object (with the two pipeline extensions mentioned above) needs to be created for each tweet. In our tests, it took over 5 seconds per 10 tweets, which is obviously impractical. Since document size does not affect the results returned by *TextRank* (Mihalcea & Tarau, 2004, p. 407), we optimized this process by running batches of 100 tweets. Frequencies of items were multiplied by the mean of the magnitudes of the tweets in the batch, as specified by the tweet’s frequency (*n*, see tweet example (1) in section "Corpus" above).

*TextRank* returns a large amount of keyword candidates, which were sorted by score, keeping those with a minimum score of 0.010 and a minimum frequency of 1 (within batches). Items from batches were aggregated by averaging scores and adding frequencies. We extracted a maximum of 500 items per day, sorting by score in the case of keywords and by frequency in the other item types (entities, emojis, hashtags, and mentions), thus keeping items with much higher scores and frequencies than the above-mentioned minimums.

We further aggregated all items by month, splitting keywords into two groups: keywords proper (single words) and keyphrases (n-grams in the range 2-4). PyTextRank does not differentiate between n-gram sizes, but we did this to facilitate comparing results with the other keyword extraction method, which returns two different sets of key items for unigrams and multi-words. Similarly, in the monthly aggregated collections, we kept 1000 single-word and 1000 multiword keywords, as this is the maximum number of keywords offered by *SketchEngine*.

To obtain the keywords with *SketchEngine*, we converted our two corpus samples from JSONL to XML format and uploaded them to the platform. *SketchEngine* allows users to create subcorpora based on several variables, such as file names and metadata. We created one subcorpus for each month (for each of the two corpora) based on the metadata we embedded in the XML exported files, and extracted the top 1000 keywords and keyphrases (“terms” in their terminology) for each month. All keywords were extracted using the English Web 2020 (enTenTen2020) corpus (Jakubíček et al., 2013) as reference corpus. We changed slightly the default *SketchEngine* settings: we set the focus parameter to 100, which according to Kilgariff (2009) will extract higher frequency words, but maintained the minimum frequency of 1. As to the identification of keywords, the attributes searched were words (as opposed to lemmas). We used these search parameters because these are the most similar to those that *TextRank* uses, as it tends to extract keywords with a higher frequency. After doing this, we compared the keywords extracted with the *TextRank* algorithm with those extracted by the reference corpus method used by *SketchEngine*.

## Results

Following the methodology described in the previous section, we obtained 24 sets of 1,000 single-word keywords and another 24 sets of multi-word keywords (keyphrases), per method of keyword extraction, i.e., a total of 96,000 items^6^.

To quantitatively summarize results, we calculated the intersections of the monthly sets of keywords produced by both methods of keyword extraction across the two samples. Table 3 contains the results aggregated by year and differentiated by type (single words and multi-words). The mean column is the key datum, which refers to the mean of the intersections of the 12 months of every year.

**Table 3:** Descriptive statistics of monthly intersections (TextRank $\cap$ SketchEngine) of sets of extracted keywords (full sets, n = 1,000 per set)

Type	Sample (%)	Year	M	SD	min	25%	50%	75%	max	M diff.^a^ (%)
Keywords	0.1	2020	35.00	2.09	31.60	33.83	35.25	36.00	38.80	
Keywords	1.0	2020	33.53	2.31	30.30	32.60	33.30	34.20	38.80	1.47
Keywords	0.1	2021	32.38	2.05	30.80	32.50	33.95	34.65	38.10	
Keywords	1.0	2021	33.82	1.67	31.30	32.52	33.55	34.75	37.20	1.44
Keyphrases	0.1	2020	24.00	2.27	20.20	22.55	23.65	25.75	27.50	
Keyphrases	1.0	2020	21.60	2.09	18.80	20.40	20.70	22.98	25.40	2.4
Keyphrases	0.1	2021	21.80	2.19	19.40	20.58	21.20	22.03	26.63	
Keyphrases	1.0	2021	21.50	2.79	17.30	20.02	20.70	22.15	27.63	0.3

“M diff.” is the difference of means between the 0.1% and the 1% samples.

As expected, single-word keywords achieve a significantly higher intersection (*M* = 33.68%, *SD* = 2.03)^7^ than keyphrases (*M* = 22.23%, *SD* = 2.34). What is more relevant to our objectives is the confirmation that there is very little difference between the shared keywords produced using the 0.1% and 1% samples, as the greatest mean difference found is 2.4% (2020 keyphrases). Thus, our quantitative analysis based on set intersection suggests that very similar results were returned by both samples despite the considerable size difference.

In order to tackle specific objective 1, a comparison of keywords extraction methods^8^, Table 4 shows the first 30^9^ global keywords for each method, sorted both by frequency and score, for the 1% sample. Several observations can be drawn from this table:

1. Multi-word expressions are prevalent in score-ranked lists, especially in the case of *TextRank*, where only 4 of the top 30 items are single words.
2. The list of frequency-ranked keywords provided by *SketchEngine* is of very poor quality, as a large proportion of the items are function words (“this”, “we”, “you”, etc.) or highly delexicalized words such as auxiliary verbs (“are”, “has”, “do”). Specifically, there are 22 such items (73.3%) and only 12 actual keywords (26.7%).
3. The score-ranked *SketchEngine* list is of better quality as it does not include any function words and all words are related to the coronavirus. However, it prioritizes certain words and phrases that intuitively should be ranked lower. Of course, this is a subjective observation that only makes sense when this list is compared to those generated by *TextRank*, but even taken in isolation, some obvious flaws stand out: (i) the list focuses excessively on vaccines (e.g., “vaccinate”, “unvaccinated”, “covid” “vaccine”, “vaccine”, “mandate”); (ii) the items in first and third position refer to a specific variant of Covid, which is in second place; (iii) the fifth-ranked item is a non-English word.
4. The keywords extracted by *TextRank* capture more accurately the contents of the corpus, but they are not without issues: while those sorted by score provide a better general view of the pandemic by covering the vaccines, the restrictions and the cases, the keywords sorted by frequency include many names of countries and US states more intensely affected by the pandemic (e.g., “china”, “india”, “america”, “florida”, “wuhan”). Conversely, the score-ranked list contains mostly multi-word expressions, as mentioned above; this, however, is simply a side effect of the post-extraction division we made, as the original PyTextRank consists of one score-ranked list of single-word and multi-word keywords.
5. One of the most important Covid-related keywords from a sociological perspective, “social distancing”, appears only in the frequency-ranked *TextRank* list (in 28^th^ position). In the *SketchEngine* lists it appears in 97^th^ position (in the score-ranked list) and in 94^th^ position (in the frequency-ranked list).

**Table 4:** Global (2020-2021) keywords sorted by frequency and score (1% sample)

	*SketchEngine*		*TextRank*	
	Ranked by Score	Ranked by Frequency	Ranked by Score	Ranked by Frequency
1	omicron	covid	covid vaccines	covid-19
2	covid	pandemic	covid cases	covid
3	omicron variant	covid-19	omicron cases	people
4	vaccinate	this	long covid	china
5	ðÿ	you	covid deaths	india
6	unvaccinated	are	covid vaccine	americans
7	covid vaccine	vaccine	covid	trump
8	delta variant	we	vaccine mandates	cdc
9	covid-19 vaccine	i	novel coronavirus	biden
10	vaccine mandate	have	covid restrictions	coronavirus
11	vax	coronavirus	pandemic	lockdown
12	lockdown	lockdown	covid patients	america
13	vaxxed	not	covid vaccination	masks
14	covid case	mask	coronavirus outbreak	florida
15	covid death	people	vaccine passports	millions
16	covid19	has	coronavirus cases	home
17	pandemic	they	vaccinated people	u.s.
18	covid-19 vaccination	do	covid mandates	vaccines
19	vaccine passport	all	coronavirus pandemic	american
20	omicron case	about	new cases	thousands
21	fauci	death	vaccine mandate	republicans
22	ivermectin	virus	people	cases
23	jab	if	covid pandemic	texas
24	covid test	who	unvaccinated people	covid19
25	vaccine	our	severe covid	wuhan
26	covid-19	trump	young people	children
27	vaccinated people	so	coronavirus	corona
28	pfizer	my	covid rules	social distancing
29	corona	no	new coronavirus	pandemic
30	vaccination	wear	coronavirus patients	deaths

The fact that “omicron” scores higher than “covid” in the *SketchEngine* score-ranked set is worth further consideration. This is quite probably due to the reference corpus that we used (enTenTen20), as the omicron variant of the SARS-CoV-2 virus came to light in November 2021. Thus, although the term “omicron” only occurs in the November and December 2021 samples, it does so with a high frequency, and thus obtains very high scores in these two months due to the comparatively low frequency it has in the reference corpus. Therefore, this case illustrates one of the main issues of the reference-corpus approach to keyword extraction: the selection of a reference corpus determines the results to a large extent.

To tackle specific objective 2, we compare the results returned by the two sample sizes in our study. Again, we calculate the intersections for the different sets of keywords and keyphrases, each set being the top 1,000 items ranked by score. Table 5 summarizes the results.

**Table 5:** Intersection percentages for the 0.1% and the 1% sample sizes

	Keywords		Keyphrases		
	2020	2021	2020	2021	
SketchEngine	84,8%	29%	66,3%	69,8%	(*M* = 62.48%, *SD* = 23.72)
***TextRank***	77,7%	83,6%	60,5%	71%	(*M* = 73.20%, *SD* = 9.91)
	(*M* = 68.78%, *SD* = 26.70)		(*M* = 66.90%, *SD* = 4.00)		

Intersection percentages are generally substantial, that is, most of the keywords were captured by both sample sizes across each of the extraction methods and morphosyntactic types (single words and multi-words). *SketchEngine* shows considerably lower intersection percentages than *TextRank* (10.72% lower on average). As suggested by the very high standard deviation (23.72), this is undoubtedly due to the extremely low intersection percentage of the 2021 keywords sets (29%). Closer examination^10^ of the differences between the two sets in question (0.1% and 1% samples) reveals that the main reason is the very high number of non-English words that this set contains: over 400 (i.e., 40%) of the keywords are foreign words, mostly in Hindi, but also in Arabic, Thai, Korean, Chinese, and others, both in their original alphabets and as Western-alphabet transcriptions. Such words, which should be excluded by the tool, are instead identified by *SketchEngine* as actual keywords and assigned a very high score.

The overall average intersection percentage (*M* = 67.84%, *SD* = 17.78), which is similar for single words and multi-words (68.78% and 66.9%, respectively), suggests that using a smaller sample size does not have a considerable impact on results, especially if we bear in mind the above-mentioned issue.

For practical purposes, however, considering 1,000 keywords is not very realistic, as we usually need much fewer in order to obtain the main topics, terms, and entities of a corpus. Table 6 lists the top 30 keywords (ranked by score) for the two sample sizes and extraction methods. Only one keyword is not present in both samples in the case of *SketchEngine* (96.7% intersection) and three in the case of *TextRank* (90% intersection). Importantly, all of the keywords in the difference are in the lowest positions in both cases.

**Table 6:** Top 30 keywords for the year 2020 by extraction method and sample size

*SketchEngine*				*TextRank*			
0.1% Sample		1% Sample		0.1% Sample		1% Sample	
Keyword	Score	Keyword	Score	Keyword	Score	Keyword	Score
coronavirus	49.32	coronavirus	47.83	coronavirus	0.75	coronavirus	0.74
covid	48.11	covid	43.75	pandemic	0.63	pandemic	0.64
covid-19	31.27	covid-19	31.40	covid	0.61	people	0.57
pandemic	31.12	pandemic	30.23	people	0.57	covid	0.55
lockdown	17.60	lockdown	18.54	covid19	0.52	covid19	0.55
virus	14.90	virus	15.16	cases	0.50	lockdowns	0.52
trump	14.12	corona	12.50	covid-19	0.47	cases	0.50
corona	11.74	trump	12.40	u.s.	0.46	case	0.48
china	11.03	ðÿ	11.33	lockdown	0.45	virus	0.46
mask	9.73	china	11.29	deaths	0.44	lockdown	0.46
deaths	9.64	mask	9.45	lockdowns	0.44	u.s.	0.46
outbreak	9.27	deaths	9.40	death	0.42	covid-19	0.45
cases	9.03	outbreak	9.28	virus	0.41	death	0.45
wuhan	9.02	cases	8.92	china	0.41	days	0.44
distancing	8.15	wuhan	8.79	masks	0.41	deaths	0.44
vaccine	7.40	distancing	8.27	lives	0.38	china	0.43
ðÿ	7.39	vaccine	7.18	trump	0.38	masks	0.43
masks	7.29	masks	7.16	home	0.37	times	0.41
covid19	6.32	covid19	5.87	case	0.37	years	0.40
wear	5.81	wear	5.63	life	0.37	health	0.40
cdc	5.69	spread	5.59	days	0.36	home	0.38
spread	5.67	cdc	5.57	health	0.36	countries	0.38
tested	5.12	stay	5.15	states	0.34	weeks	0.38
americans	5.01	novel	5.01	years	0.34	mask	0.37
stay	4.97	tested	4.71	mask	0.33	life	0.37
positive	4.64	flu	4.54	weeks	0.33	lives	0.37
chinese	4.47	chinese	4.46	**patients**	0.33	states	0.36
**breaking**	4.46	positive	4.46	government	0.32	trump	0.36
infected	4.39	infected	4.44	**hospitals**	0.31	government	0.35
flu	4.35	americans	4.35	**corona**	0.31	state	0.35

**Fig. 2:**
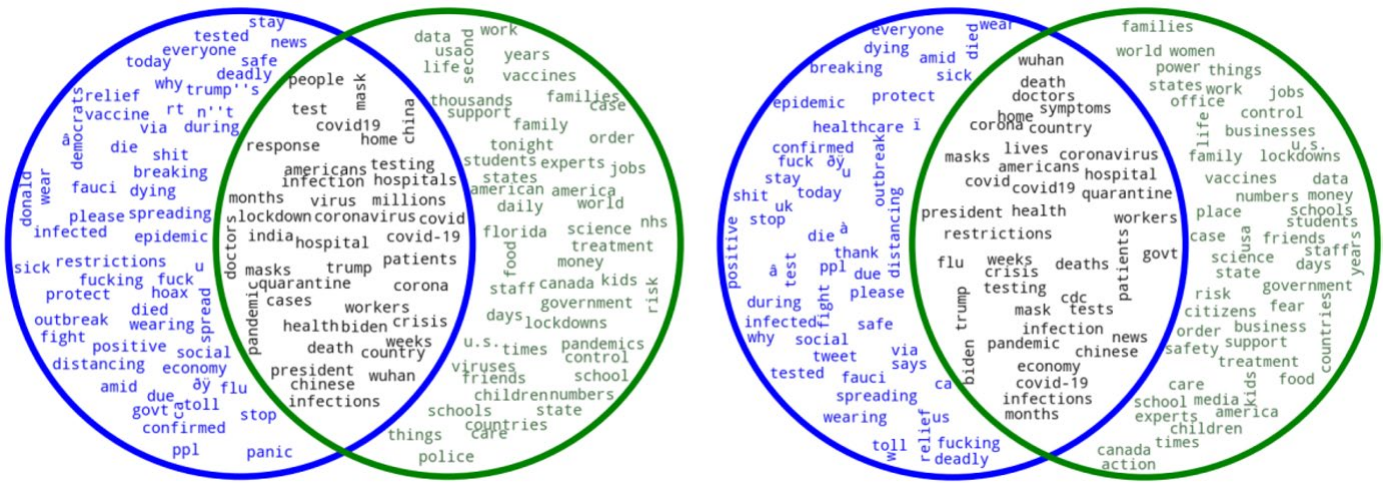
Keywords obtained by SketchEngine and TextRank in 2020 (0.1% left, 1% right; SE in blue, TR in green)

Finally, in order to compare extraction methods along with sample size in a more detailed way, we generated Venn diagrams with word clouds. Figure 2 shows two Venn diagrams that summarize the keywords obtained by *SketchEngine* and *TextRank* in 2020^11^.

**Fig. 3:**
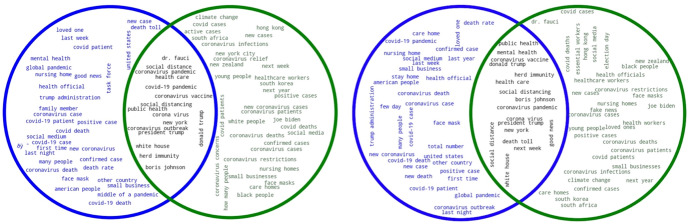
Keyphrases obtained by SketchEngine and TextRank in 2020 (0.1% left, 1% right; SE in blue, TR in green)

The figure shows the results for the 0.1% (left), and those for the 1% sample (right). In both cases the keywords obtained by *SketchEngine* are shown in blue, while keywords provided by *TextRank* are in green. The intersection, representing the keywords that both systems have extracted, is shown in black. Figure 3 also shows two Venn diagrams that summarize the keyphrases obtained for both sample sizes.

As to the keywords shown in Fig. 2, in both cases the intersection shows very similar results: keywords mainly associated with the pandemic throughout 2020 and which seem to summarize the main events that took place worldwide during that year (“*Wuhan*”, “*symptoms*”, “*quarantine*”, “*infection*”). Some of these words were repeated (e.g., “hospital” and “hospitals”) because the corpus was no lemmatized. We can also find variants of terms that refer to the same concept, such as “covid”, “covid19”, and “covid-19”.

However, the intersection goes beyond the main general consequences of the pandemic, as it is possible to find key political figures (i.e., Donald Trump and Joe Biden) in both sample sizes as a consequence of the 2020 United States presidential elections. As shown in example (2), Twitter users talked about the presidential candidates within the COVID-19 context.

(2) I think Joe **Biden** should NOT stop campaigning just because **Trump** was stupid enough not to follow COVID-19 guidelines and “catch it.”

Although the keyword “quarantine” appears in both intersections, in closer examination we noticed that it stopped being relevant after August 2020. The reason is that most countries went into lockdown between March 2020 and July 2020. In the case of the United Kingdom, for example, on July 4^th^ 2020, the Health Protection Regulations 2020^12^ came into force, which entailed the relaxation of the previous Lockdown Regulations. Consequently, there were fewer restrictions, and businesses such as cafes, bars, and museums (to name a few) reopened. In this sense, it is only logical that by August 2020 this word had stopped being as relevant as before. Despite this, people were still being quarantined if they had COVID symptoms, hence the importance of this word in the corpus, as in (3):

(3) A school district in Georgia reported today that 260 employees have tested positive for the coronavirus or are in **quarantine** because of possible exposure as they prepare for the new school year.

More examples also show the relevance of this keyword in relation to travel restrictions, since travelers were in many cases forced to quarantine in their destinations, as shown in (4):

(4) Really wanting the Government to take away the 14 day **quarantine** rule for returning from Spain  really want to go and visit my parents out there

In addition, we do find differences between the two sample sizes. For example, the following keywords were not included in the 1% sample, but did appear in the 0.1%: “china”, “india”, “lockdown”, “millions”, “people”, “response”, “test”, and “virus”. Therefore, the 1% sample fails to include relevant terms that are part of the main aspects of the pandemic, such as “*lockdown*”, “*test*”, or “*virus*”. In the case of the 0.1% sample, the following keywords were missing: “cdc”, “economy”, “flu”, “gvt”, “lives”, “news”, “restrictions”, and “symptoms”. In spite of these differences, however, the intersection of both sample sizes for 2020 still represents many of the main events associated with the pandemic appropriately and both present generally similar results.

As for the keywords extracted by the two different systems, *SketchEngine* returns many wrongly decoded Unicode characters from languages other than English (mostly Hindi) that should not be counted as keywords, because they simply add noise to the extraction method, such as “â”, “à”, “w”, and “ï”. In fact, the bigger the sample, the higher the number of such characters, as can be seen in Fig. 2. This, however, is not the case with *TextRank*, which only provides actual words that can be considered keywords.

**Fig. 4:**
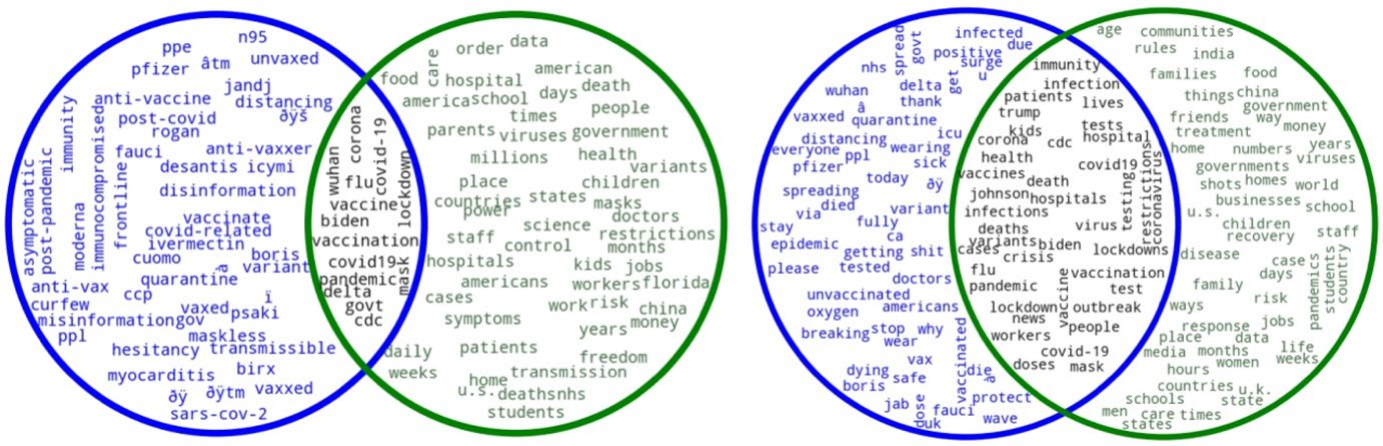
Keywords obtained by SketchEngine and TextRank in 2021 (0.1% left, 1% right; SE in blue, TR in green)

**Fig. 5:**
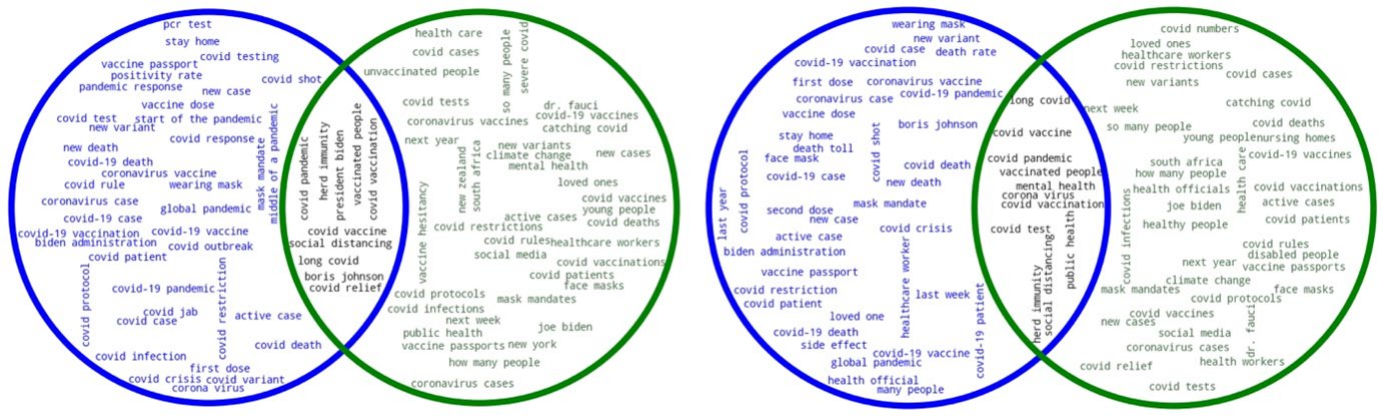
Keyphrases obtained by SketchEngine and TextRank in 2021 (0.1% left, 1% right; SE in blue, TR in green)

In the case of the keyphrases, the intersections of both sample sizes show very similar results: some of the main general events and measures related to the pandemic (*coronavirus pandemic*, *social distance*, *coronavirus vaccine*) are, once again, intertwined with references to politics (Dr. Fauci, President Trump, White House, Boris Johnson, Donald Trump). As for the keyphrases obtained by *TextRank*, these merge references to the pandemic with phrases pointing to social (rather than medical) aspects of the pandemic, such as “first time”, “last week”, “family member”, or “social medium”. *TextRank* returns actual keyphrases, but also includes some that, at first sight, might be deemed as unimportant (“New York City”, “South Africa”, “Hong Kong”, and “South Korea”). Nevertheless, such proper nouns indicating locations are actually relevant in this corpus, as they refer to some of the cities that struggled the most with the virus. Figures 4 and 5 summarize the keywords and keyphrases obtained by *SketchEngine* and *TextRank* for 2021.

The results for the 2021 keywords vary greatly according to the sample size. In both cases, the intersection includes some of the main events, broadly speaking, that took place during 2021 (“vaccine”, “delta”, “mask”). In the case of the 0.1% sample, however, the number of words that both extraction methods share is much lower if we compare it to the 1%. Thus, the intersection from the 1% sample covers a wider range of keywords that, in a general sense, summarize some of the main events that characterized 2021 from the perspective of the pandemic, many of them referring to the vaccination process (e.g., “vaccines”, “doses”, and “immunity”). This can be seen in examples (5) and (6) below:

(5) The key to immunity is **vaccination**, not infection. RT if you agree.
(6) The shot is required to help your body build **immunity**. You can still carry the virus with the shot you just will not have symptoms this is why we are still wearing masks

The 2021 keyword intersection of the 1% sample also reflects the different COVID-19 variants that appeared this year, such as Omicron, Delta, Theta, and Zeta (European Centre for Disease Prevention & Control, 2022). The presence of such variants led Twitter users, as can be seen in (7), to express their concerns:

(7) The #Delta**Variant** is more dangerous than other **variants** of the virus that causes #COVID19. Get vaccinated as soon as you can.

As for the key political figures, the intersection of the 0.1% sample only includes Joe Biden (8), whereas that of the 1% also includes Boris Johnson (9) and Donald Trump (10).

(8) This is supposed to change my point how? **Biden** put forward a similarly insufficient covid relief
(9) Beyond ironic, given **Johnson** is using Covid to accelerate NHS privatisation. Biting the hand that cared for you...
(10) President **Biden** is focused solely on helping the American people after Donald **Trump** did nothing about a deadly pandemic.

Nevertheless, the “johnson” keyword also refers to the company Johnson & Johnson, supplier of COVID-19 vaccines and whose doses resulted in numerous cases of blood clots and other secondary effects:

(11) More blood clot cases following **Johnson** and **Johnson** COVID-19 vaccine reported

Although during 2021 COVID-19 lockdowns were not as equally widespread around the world as in 2020, countries such as Australia still went into lockdown during that year (Knowlton, 2022). This is reflected in both sample sizes, and, as can be seen in example (12), Twitter users published their tense responses to these measures:

(12) “There are new variants, we have to **lockdown** again.” There are new variants of every virus every year. If we continue to accept this, we are never escaping this.

In conclusion, the intersection of keywords obtained during 2021 varies greatly according to the sample, which highlights the importance of choosing the appropriate size according to the needs of our research. Also, as in the 2020 sample, *SketchEngine* returns wrongly decoded Unicode characters that should not be counted as keywords, such as “ï” or “ðÿs”, among others. This, again, is not the case of *TextRank*, which only returns actual words.

It is also relevant to point out that the keywords provided by *TextRank* in 2020 are more COVID-related, while in 2021 these seem to be more general. Thus, there is a higher degree of variability for *TextRank* between 2020 and 2021, which is not the case for *SketchEngine*. In fact, *SketchEngine* keywords provide higher insight in terms of the events that took place during the pandemic and how it evolved in time. Some of these keywords are “curfew*”* (13) and “myocarditis” (14).

(13) Miami Beach officials impose Covid **curfew** to curb spring break chaos.
(14) Oregon Health Authority said aware of at least 11 cases of **myocarditis** or pericarditis following COVID vaccination, including 15-year old boy hospitalized after receiving second dose of vaccine.

*SketchEngine* also identifies *unvaccinated* as a keyword. The vaccination process characterized 2021, as it was during that year that the vaccines began to be produced and the population around the world had access to them. As shown in example (15), users often talked about the importance of being vaccinated by referring to the long-time risks of being unvaccinated.

(15) The willful ignorance here, ignoring the fact that Covid spreads because **unvaccinated** people carry it and spread it, and that if you are **unvaccinated** and get Covid your chances of having long Covid or dying are higher.

The intersection of the 2021 keyphrases shows that these revolve around the pandemic with a more specific focus than the keyphrases obtained for 2020: “long covid”, “herd immunity” and the “covid vaccine” appear in both sample sizes. Nevertheless, it must be noted that while references to politics appear in the 0.1% sample (“President Biden”, “Boris Johnson”), these are not present in the 1% sample. Regarding the keyphrases obtained by each extraction method, both *SketchEngine* and *TextRank* provide more accurate results from the 2021 sample, as they only extract expressions that are related to the pandemic.

## Conclusions

The results of this study show that keyword extraction is a valuable resource for the exploration of large social media corpora, as it provides a clear pathway and an entry point for the qualitative researcher into an otherwise intractable mass of data. The use of multiple keyword extraction methods, such as *TextRank* and *SketchEngine* in our work*,* provides interesting results, as the nature of the keywords extracted varies. Frequency-based methods that employ a reference corpus (as exemplified by *SketchEngine*) have the non-trivial issue derived from the need to select one particular corpus, as words in the focus corpus that have low frequency in the reference corpus will rank high in the keywords list. On the other hand, the keywords extracted by graph-based methods (as exemplified by *TextRank*) seem to capture more accurately the general contents of the corpus.

Thus, the analysis of the keywords per year shows that the results obtained by *TextRank* seem to better capture the nature of the corpus, while those provided by *SketchEngine* are more content-specific and depend on the search parameters employed. Ultimately, these two keyword extraction methods may be appropriate for different aims: graph-based methods seem to be more appropriate to retrieve the most salient topics, events, and entities of a corpus, while statistics-based methods are better at extracting specialized terms. Despite this, the results provided by each method must not be understood as opposed to each other but rather as complementary, as the keywords in the intersection between both systems could be considered to thoroughly represent not only the main events and concepts of the pandemic in a general sense, but also relevant aspects of politics that are also related. There is, however, a higher degree of variability for *TextRank* according to the year under study: in 2020 the keywords obtained were more COVID-related, while in 2021 these became more general, which is not the case of *SketchEngine*. The keywords obtained by *SketchEngine*, on the other hand, provide good insights about the events that took place during the pandemic. Finally, *SketchEngine* blindly returns high-frequency non-English words, an issue that is not present in *TextRank*.

As for sample sizes, our study suggests that, with very large social media corpora, smaller samples produce comparable results to bigger ones. Thus, when dealing with very large social media corpora, it may be unnecessary to extract larger samples for the sake of statistical representativeness, as smaller samples can also be both representative and relevant for the qualitative researcher, as well as easier to process from a computational perspective.

## Data Availability

The original corpus by Chen et al. (2020) is freely available and can be found at https://github.com/echen102/COVID-19-TweetIDs. All of our datasets are publicly available on https://github.com/Diverking/COVID-19.

## Footnotes

1 “There’s no data like more data” is a quote coined by Robert Mercer of the IBM Continuous Speech Recognition Group in 1988. It has become a classic quote in the fields of Artificial Intelligence, Machine Learning, and Data Science.

2 For example, AntConc (Anthony, 2022) offers a choice of chi-squared and log likelihood statistics, and several options for statistic threshold, effect size measure, and effect size threshold.

3 The dataset is freely available and can be found at https://github.com/echen102/COVID-19-TweetIDs.

4 By “accessible” we mean without resorting to indexing algorithms or advanced database management systems. In terms of hardware, we employed conventional, mid-range non-GPU systems.

5 This extension is available on https://github.com/explosion/spacymoji.

6 All our datasets are publicly available on https://github.com/Diverking/COVID-19.

7 The mean of means is acceptable here, since all subsets have exactly the same sample size (n = 1000).

8 It is important to bear in mind that, although we will be referring to these two methods as *TextRank* and *SketchEngine*, in fact we are comparing graph-based methods (embodied by the former) with statistical, reference corpus-based methods (embodied by the latter).

9 The full list can be found in the article’s data repository.

10 The full results of the analysis, including the lists of intersections and differences can be found in article’s repository under the folder “sample compare”.

11 Venn diagrams are convenient to visualize this type of data, but they have limitations in terms of the number of items that can be displayed, as either the size of the font is too small or the size of the figure is too large. After several tests, we compromised on 200 items per diagram (100 for each keyword extraction method). Thus, it is important to understand that the results discussed below refer exclusively to the top 100 keywords (sorted by score) returned by each method for each year, which is obviously a substantial reduction of the original datasets. To overcome this limitation, we have included the full intersection lists as tables in HTML documents in the article’s Github repository mentioned above. For each sample size, we include 8 HTML files, 4 for each year: 2 for keywords (sorted by score and frequency) and 2 for keyphrases.

12 The Health Protection (Coronavirus, Restrictions) (No. 2) (England) Regulations 2020 (SI 2020/684).
